# β Subunits Functionally Differentiate Human Kv4.3 Potassium Channel Splice Variants

**DOI:** 10.3389/fphys.2017.00066

**Published:** 2017-02-08

**Authors:** Geoffrey W. Abbott

**Affiliations:** Bioelectricity Laboratory, Department of Pharmacology and Department of Physiology and Biophysics, School of Medicine, University of California, IrvineIrvine, CA, USA

**Keywords:** Brugada syndrome, cardiac arrhythmia, KChIP, KCNE, potassium channel

## Abstract

The human ventricular cardiomyocyte transient outward K^+^ current (*I*_to_) mediates the initial phase of myocyte repolarization and its disruption is implicated in Brugada Syndrome and heart failure (HF). Human cardiac *I*_to_ is generated primarily by two Kv4.3 splice variants (Kv4.3L and Kv4.3S, diverging only by a C-terminal, S6-proximal, 19-residue stretch unique to Kv4.3L), which are differentially remodeled in HF, but considered functionally alike at baseline. Kv4.3 is regulated in human heart by β subunits including KChIP2b and KCNEs, but their effects were previously assumed to be Kv4.3 isoform-independent. Here, this assumption was tested experimentally using two-electrode voltage-clamp analysis of human subunits co-expressed in *Xenopus laevis* oocytes. Unexpectedly, Kv4.3L-KChIP2b channels exhibited up to 8-fold lower current augmentation, 40% slower inactivation, and 5 mV-shifted steady-state inactivation compared to Kv4.3S-KChIP2b. A synthetic peptide mimicking the 19-residue stretch diminished these differences, reinforcing the importance of this segment in mediating Kv4.3 regulation by KChIP2b. KCNE subunits induced further functional divergence, including a 7-fold increase in Kv4.3S-KCNE4-KChIP2b current compared to Kv4.3L-KCNE4-KChIP2b. The discovery of β-subunit-dependent functional divergence in human Kv4.3 splice variants suggests a C-terminal signaling hub is crucial to governing β-subunit effects upon Kv4.3, and demonstrates the potential significance of differential Kv4.3 gene-splicing and β subunit expression in myocyte physiology and pathobiology.

## Introduction

Human voltage-gated potassium (Kv) channels are generated by a gene family expressing 40 different α subunits, producing a wide range of current properties and diversity of conductances, gating kinetics and voltage dependence. Kv channels exist as tetramers that form a functional pore, and channel function can be diversified in some cases by formation of heteromeric channels bearing more than one type of α subunit, typically within the same Kv subfamily (Xu et al., 1995). Another mechanism for amplification of this diversity is splice variation (Vega-Saenz de Miera et al., 1992).

Kv4 subfamily α subunits (Kv4.1, Kv4.2, and Kv4.3), encoded by three genes (*KCND1, 2*, and *3*, respectively), generate subthreshold-activating Kv currents that are transient because they exhibit fast inactivation, causing current decay over tens to hundreds of milliseconds (Serôdio et al., 1994, 1996). Kv4.3 is strongly expressed in human cardiomyocytes and neurons. In neurons, Kv4.3 contributes to *I*_SA_ (Serôdio et al., 1994), while in cardiac myocytes it generates *I*_to_ (transient outward current; Dixon et al., 1996; Johns et al., 1997), important for early myocyte repolarization to counteract depolarization caused by cation influx through voltage-gated sodium and calcium channels. Kv4.1, 2, and 3 exhibit close sequence alignment, except for a divergent additional exon in Kv4.3, beginning after residue 487, encoding 19 amino acids within the S6-proximal cytosolic C-terminus (residues 488–506) and unique to Kv4.3. This additional exon is commonly spliced such that two Kv4.3 splice variants exist in human tissues. The short version (Kv4.3S) lacks the 19 residues whereas the long form (Kv4.3L) possesses the additional 19 amino acids but is otherwise identical to Kv4.3S (Ohya et al., 1997; Kong et al., 1998).

Both forms of Kv4.3 are expressed in human and rat heart (Ohya et al., 1997; Kong et al., 1998) but their expression levels are altered differentially in human heart failure (HF), Kv4.3L expression rising ~33% while that of Kv4.3S falls ~75% (Radicke et al., 2006). HF has reached epidemic proportions, with a prevalence of >37.7 million people worldwide, and almost 6 million in the United States alone, half of whom die within 5 years of diagnosis. HF occurs when heart muscle becomes so weak that it cannot adequately pump blood into the aorta to supply the body with oxygenated blood, resulting in pooling of blood in the heart. HF risk increases with diabetes, obesity, hypertension and coronary artery disease, and lifestyle factors including a sodium- and cholesterol-rich diet, and lack of exercise (Roger, 2013; Mozaffarian et al., 2016; Ziaeian and Fonarow, 2016). HF is tightly associated with reduced *I*_to_ density and Kv4.3 expression in human and canine ventricular myocytes. A reduction in *I*_to_ alters cardiomyocyte action potential morphology and duration, particularly the early repolarization and plateau phases, at least partially by perturbing voltage-gated Ca^2+^ channel activity (Beuckelmann et al., 1993; Näbauer et al., 1993; Kääb et al., 1998; Zicha et al., 2004). Approximately half of individuals with HF die suddenly, with ventricular tachyarrhythmias being a likely cause in many cases (Cohn et al., 1986; Stevenson et al., 1993). A greater understanding of the processes underlying perturbation in HF of ionic currents, including *I*_to_, known to be associated with ventricular tachyarrhythmias in other genetic arrhythmia syndromes, has the potential to lead to therapeutic advances in reducing morbidity and mortality in HF.

Thus, the prior finding that HF splice-dependently remodels Kv4.3 in human heart is potentially of high significance. However, homomeric Kv4.3L and Kv4.3S are functionally indistinguishable at baseline, in terms of macroscopic current density, gating kinetics, and voltage dependence. Subtle isoform-dependent differences arise because Kv4.3L contains a consensus phosphorylation site in its unique 19-residue stretch. Protein kinase C (PKC) phosphorylation of threonine 504 within this site differentially modulates Kv4.3L closed state inactivation, increasing its magnitude in Kv4.3L, while in Kv4.3S (lacking this site) PKC reduces closed state inactivation. Aside from this, other effects of PKC were found to be Kv4.3 isoform-independent, similarly inhibiting peak current and not altering open-state inactivation or recovery from open-state inactivation (Xie et al., 2009).

Kv currents are further diversified *in vivo* by formation of macromolecular complexes between Kv α subunits and other proteins that alter their functional characteristics. Many of these channel-regulating proteins appear to be *bona fide* channel components, forming stable, obligate, heteromeric channel complexes with α subunits *in vivo*, and are referred to as ancillary or β subunits (in the case of Kv channel modulators). Two major classes of Kv channel β subunits expressed in human heart are the KCNEs and the K^+^ channel interacting proteins (KChIPs; Abbott and Goldstein, 1998; An et al., 2000). The KCNEs (also termed MinK-related peptides, or MiRPs) are single-transmembrane-domain subunits that co-assemble with Kv α subunits to dictate fundamental properties including channel α subunit composition, forward trafficking, endocytosis, ion selectivity, conductance, gating kinetics, voltage dependence, and the effects of regulation by other proteins (Abbott, 2015, 2016a,b). KChIPs are cytosolic proteins that also modulate multiple aspects of Kv channel biology, including trafficking, current density, gating kinetics, and voltage dependence (An et al., 2000; Rhodes et al., 2004).

Kv4 channels are able to co-assemble with KCNE proteins and with KChIPs, almost certainly at the same time, forming heteromeric channels with varied gating properties and current magnitude (Radicke et al., 2006; Liu et al., 2008; Levy et al., 2010). It is thought that Kv4.3 is regulated in human heart by KChIP2 (and in particular the dominant cardiac isoform, KChIP2b) and one or more of the KCNEs (Kuo et al., 2001; Deschênes and Tomaselli, 2002; Radicke et al., 2006). Indeed, cardiac arrhythmias including Brugada syndrome have been associated with increased ventricular *I*_to_ arising from gain-of-function mutations in *KCND3* (Giudicessi et al., 2011), but also in *KCNE3* (Delpón et al., 2008) and *KCNE5* (Ohno et al., 2011). Despite a plethora of studies on Kv4.3 regulation by KCNEs and KChIPs on the one hand, and of the function and expression of Kv4.3L vs. Kv4.3S on the other hand, a comparison of how the two Kv4.3 splice variants respond to regulation by β subunits has been lacking, and this response was assumed to be isoform-independent (Radicke et al., 2006). Here, it is demonstrated that KChIP2b and KCNEs unexpectedly introduce a number of striking differences in baseline function between human Kv4.3L and Kv4.3S. These data both identify a 19-residue C-terminal stretch important for Kv4.3 regulation by β subunits, and suggest the functional and pathophysiological significance of differential remodeling of Kv4.3 splice variants in HF.

## Materials and methods

### *Xenopus laevis* oocyte channel cRNA and peptide injections

cRNA transcripts encoding hKv4.2, hKv4.3L, hKv4.3S, and hKChIP2b were generated by *in vitro* transcription (T7 polymerase mMessage mMachine kit, Thermo Fisher Scientific) from cDNA sub-cloned into plasmids (a kind gift of Dr. Steve A. N. Goldstein) incorporating *Xenopus laevis* β-globin 5′ and 3′ UTRs flanking the coding region to enhance translation and cRNA stability, after vector linearization. Human KCNE genes 1–5 [for KCNE3 and KCNE4, the recently cloned full-length “L” versions (Abbott, 2016c) were used] were similarly transcribed from cDNA templates also incorporating *Xenopus laevis* β-globin 5′ and 3′ UTRs. cRNA was quantified by spectrophotometry. Defolliculated stage V and VI *Xenopus laevis* oocytes (Ecocyte Bioscience, Austin, TX) were injected with one, two or three of the subunit cRNAs as follows: 0.04–5 ng of Kv4.x; 0.5–5 ng of KChIP2b, 5 ng of KCNEx (or 1 ng in some batches for KCNE5, to minimize oocyte toxicity), as indicated, per oocyte. Oocytes were incubated at 16°C in SBB solution (Ecocyte) containing penicillin and streptomycin, with daily washing, for 36–48 h prior to two-electrode voltage-clamp (TEVC) recording. For peptide studies, synthetic 19-mer peptides corresponding to the Kv4.3L-specific C-terminal 19-residue stretch (“L peptide”; GLSYLVDDPLLSVRTSTIK; 99.2% pure) or a scrambled control peptide (VLDRLSLYTLIPSKTGVDS; 98.5% pure) were commercially synthesized (Peptide 2.0, Chantilly, VA, USA). Peptides were solubilized to 500 μM in an aqueous solution containing (in mM) 96 KCl, 2 NaCl, 1 MgCl_2_, 1 CaCl_2_, 10 HEPES (pH 7.6), and 50 nl injected into oocytes 30–90 min before TEVC recording to give an estimated final intracellular peptide concentration of 25 μM (assuming a mean oocyte volume of 1 μl).

### TEVC

TEVC recording was performed at room temperature with an OC-725C amplifier (Warner Instruments, Hamden, CT) and pClamp8 software (Molecular Devices, Sunnyvale, CA), on *Xenopus* laevis oocytes placed in a small-volume oocyte bath (Warner), visualized with a dissection microscope. The TEVC bath solution was (in mM): 96 NaCl, 4 KCl, 1 MgCl_2_, 1 CaCl_2_, 10 HEPES (pH 7.6); bath chemicals were from Sigma. TEVC pipettes were of 1–3 MΩ resistance when filled with 3 M KCl. Potassium ion currents were recorded in response to a Voltage protocol consisting of pulses between −80 and +60 mV at 20 mV intervals, from a holding potential of −80 mV, to yield current-Voltage relationships and for fitting of inactivation kinetics. For quantification of steady-state inactivation, oocytes were held at −100 mV and prepulsed to Voltages between −120 and 0 mV followed by a tail pulse to +40 mV. For quantification of inactivation recovery rates, oocytes were double-pulsed to +40 mV with variable recovery times (10–5000 ms) at −120 mV between pulses, and the magnitude of the second peak compared to that of the initial peak for each pair. A generic human ventricular action potential Voltage protocol was also utilized for comparing effects of channel subunit expression changes; the protocol is shown in **Figure 9**.

TEVC data analysis was performed with Clampfit (Molecular Devices) and Origin 6.1 (OriginLab Corp., Northampton, MA) software. Values are stated as mean ± SEM. Steady-state inactivation plots of fraction of available channels vs. Voltage were plotted vs. prepulse voltage and fitted with a single Boltzmann function according to:

(1) $$g=\left({\text{A}}_{1}-{\text{A}}_{2}\right)/\left\{1+\text{exp}\left[\left({\text{V}}_{1/2}-\text{V}\right)/{\text{V}}_{\text{s}}\right]\right\}+A_{2}$$

where *g* is the normalized tail conductance, A_1_ is the initial value at −∞, A_2_ is the final value at +∞, V_1/2_ is the half-maximal voltage of activation, and V_s_ the slope factor. Current decay arising from channel inactivation curves was fitted with a standard (zero-shift) single (where possible) or double exponential decay function with Chebyshev 4-point smoothing filter. Inactivation recovery kinetics were fitted from mean normalized fractional recovery currents to a two-phase exponential association equation:

(2) $$\begin{array}{c} \text{y}={\text{y}}_{0}+{\text{A}}_{1}\left(1-{\text{e}}^{-{\text{x}/\text{t}}_{1}}\right)+{\text{A}}_{2}\left(1-{\text{e}}^{-{\text{x/t}}_{2}}\right) \end{array}$$

and for cases in which iterative fitting yielded identical τ-values, a single exponential fit was reported. Because mean inactivation recovery curves were fitted to improve fit, these data are reported as a value with no standard error, but rather a chi-squared test for goodness of fit. In all other cases, values are reported with standard error of the mean. Where informative, currents were compared with one another using student's *t*-test to assess statistical significance (*P* < 0.05). For consistency, current magnitudes were always compared between groups at a membrane potential of +40 mV. If multiple comparisons were performed, a *post-hoc* Tukey's HSD-test was performed following ANOVA.

## Results

### KChIP2b is a more potent augmenter of Kv4.3S vs. Kv4.3L current

**Figure 1:**
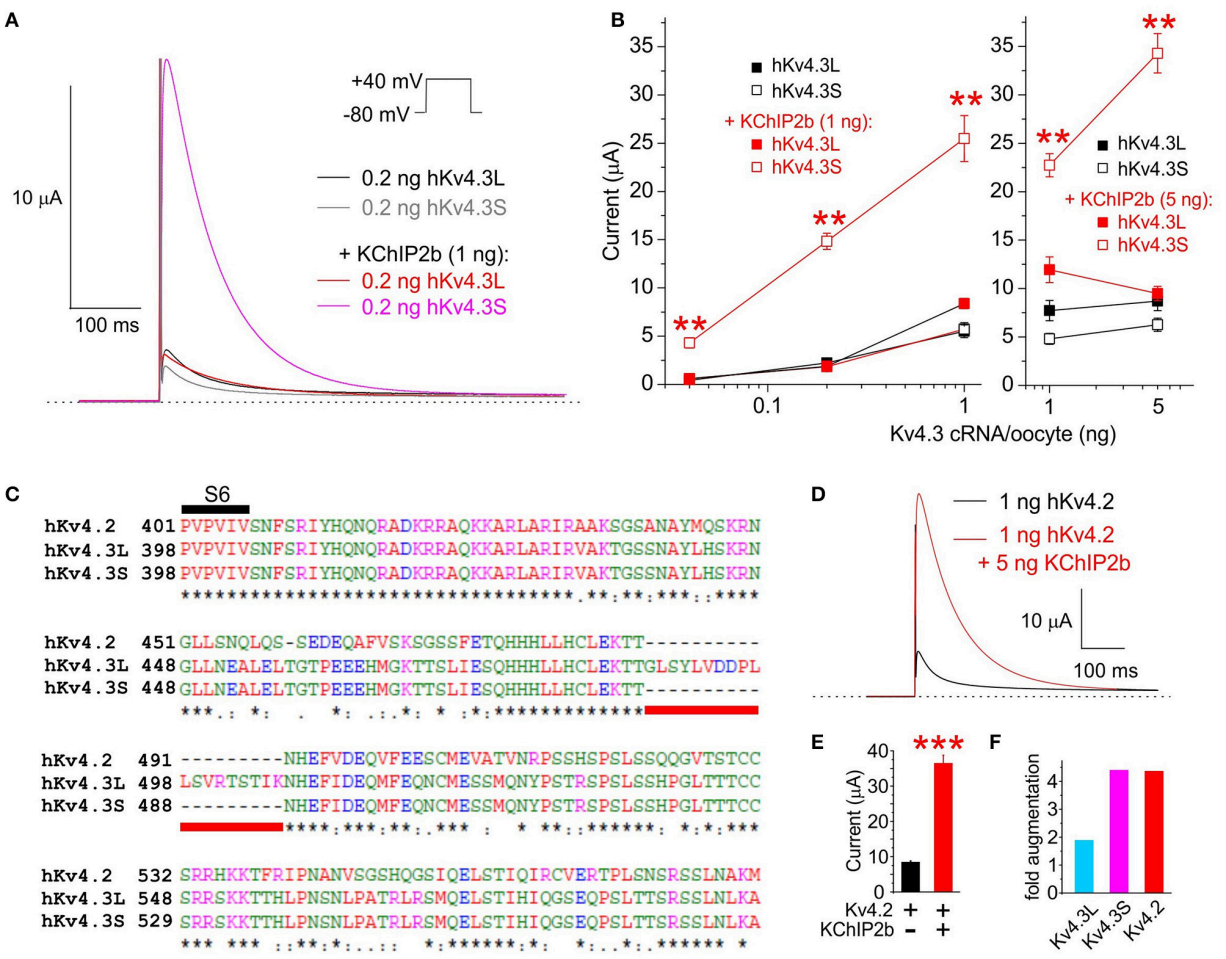
**KChIP2b differentially augments Kv4.3L and Kv4.3S current magnitude. (A)** Exemplar current traces recorded at +40 mV (voltage protocol inset) from *Xenopus* oocytes 36–40 h after injection of 0.2 ng cRNA encoding Kv4.3L or Kv4.3S, with or without 1 ng KChIP2b (*n* = 14–18). *Insets*: *left*, scale bars; *right*, Voltage clamp protocol. Zero current level indicated by dashed line. **(B)** Mean ± SEM peak current magnitude at +40 mV for currents recorded as in **(A)** but with 0.04–5 ng Kv4.3L or S cRNA, with or without 1 or 5 ng KChIP2b cRNA, injected per oocytes, as indicated (*n* = 14–37). ^**^P < 0.01 vs. all other groups with similar quantity of cRNA injected, by ANOVA followed by Tukey's HSD test. **(C)** Sequence alignment (Clustal TCoffee) of human Kv4.2, Kv4.3L and Kv4.3S protein sequences in the S6 to C-terminal region, including the segment missing in Kv4.3s (underlined red). **(D)** Exemplar current traces recorded at +40 mV (voltage protocol as in panel A) from *Xenopus* oocytes 36–40 h after injection of 1 ng cRNA encoding hKv4.2 with or without 5 ng hKChIP2b cRNA (*n* = 16–18). Scale bar inset. Zero current level indicated by dashed line. **(E)** Mean ± SEM peak current magnitude at +40 mV for currents recorded as in **(E)** (*n* = 16–18). ^***^P < 0.0001 between groups. **(F)** Comparison of Kv4.x current augmentation by hKChIP2b, analyzed from data in panels B and F (1 ng Kv4.x, 5 ng hKChP2b cRNA; *n* = 16–37).

TEVC analysis indicated that KChIP2b, the predominant KChIP isoform in human cardiac myocytes, augments Kv4.3S activity at +40 mV several-fold more than that of Kv4.3L, across a >100-fold range of Kv4.3 cRNA quantity injected per oocyte. At 0.2 ng Kv4.3 cRNA per oocyte, co-injection of 1 ng KChIP2b augmented Kv4.3S current >8-fold but had negligible effects on Kv4.3L current magnitude (Figures 1A,B). Similar to hKv4.3S, hKv4.2 lacks the 19-residue C-terminal portion unique to Kv4.3L (Figure 1C). Also similar to hKv4.3S, hKv4.2 current at +40 mV was augmented >4-fold by hKChIP2b, compared to <2-fold for hKv4.3L (1 ng Kv4.x and 5 ng KChIP2b cRNA injected per oocyte; Figures 1D–F). Thus, the 19-residue stretch unique to Kv4.3L impairs Kv4 current augmentation by KChIP2b.

### KChIP2b differentially regulates inactivation of Kv4.3S vs. Kv4.3L

**Figure 2:**
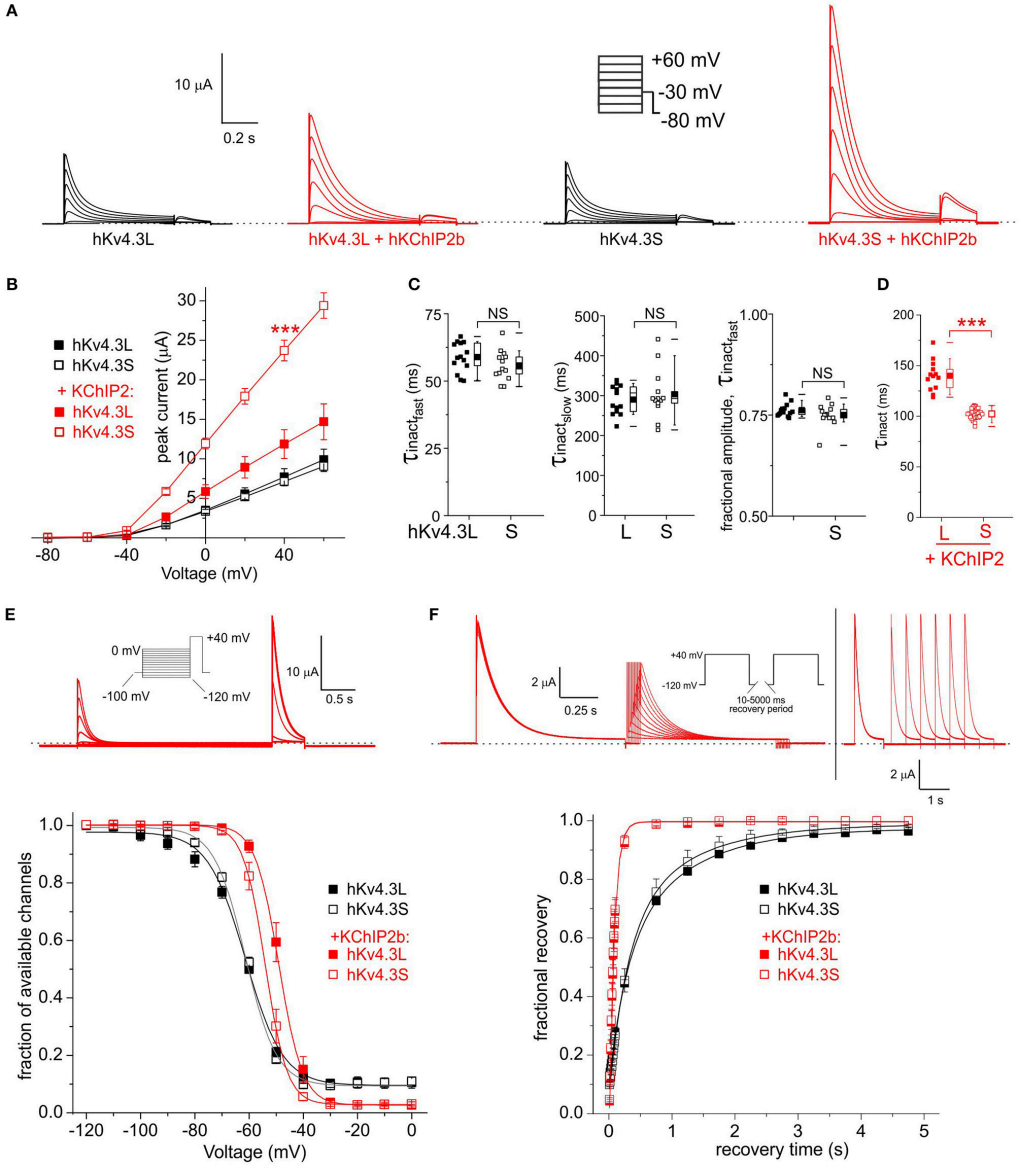
**KChIP2b differentially affects Kv4.3L and Kv4.3S inactivation rate and voltage dependence. (A)** Exemplar current traces recorded from *Xenopus* oocytes 36–48 h after injection of 1 ng cRNA (as indicated) encoding Kv4.3L or Kv4.3S alone (black, *n* = 13–14) or in addition to 5 ng cRNA encoding KChIP2 (red, *n* = 13–31). *Insets*: *right*, Voltage clamp protocol; *left*, scale bars. Zero current level indicated by dashed line. **(B)** Mean ± SEM peak current/voltage relationship for currents as in **(A)**; *n*-values as in **(A)**. **(C)** Box plots showing individual and mean ± SEM values for fast and slow τ of inactivation, and relative amplitude of the fast component (double exponential fit), of Kv4.3L vs. Kv4.3S, recorded as in **(A)**. *n* = 13–14. NS, *P* > 0.05. **(D)** Box plots showing individual and mean ± SEM values for τ of inactivation (single exponential fit) of Kv4.3L vs. Kv4.3S, with KChIP2 co-expression. Currents recorded as in **(A)**; *n* = 13–14. ^***^P < 1 × 10^−15^. **(E)** *Upper*, exemplar current trace recorded using a steady-state inactivation protocol, from *Xenopus* oocytes 36–48 h after injection of cRNA encoding Kv4.3L (1 ng) and KChIP2 (5 ng). *Insets*: *left*, Voltage clamp protocol; *right*, scale bars. Zero current level indicated by dashed line. *Lower*, mean ± SEM fraction of available channels/voltage relationship for Kv4.3L vs. Kv4.3S, with/without KChIP2b; currents recorded as in **(E)**; *n* = 6–8. **(F)** *Upper*, exemplar current trace recorded using an inactivation recovery protocol, from *Xenopus* oocytes 48 h after injection of cRNA encoding Kv4.3L (1 ng) and KChIP2 (5 ng). *Insets*: *left* and *lower right*, scale bars; *center*, voltage clamp protocol. Zero current level indicated by dashed line. *Lower*, mean ± SEM fractional recovery from inactivation/recovery time for Kv4.3L vs. Kv4.3S, with/without KChIP2b; recorded as in protocol above; *n* = 4–6.

For subsequent experiments, unless otherwise indicated, 1 ng Kv4.3 cRNA per oocyte was injected, with or without 5 ng per oocyte KChIP2b and/or KCNE cRNA, to favor saturation of channels with β subunits while also generating large enough currents for accurate analysis. As previously reported (Po et al., 2001), homomeric Kv4.3L current magnitude was indistinguishable from that of homomeric Kv4.3S across the −80 to +60 mV voltage range. In contrast, KChIP2b augmented Kv4.3S more efficiently than Kv4.3L across the voltage range tested (Figures 2A,B). Similar to previous reports (Po et al., 2001), homomeric Kv4.3L and Kv4.3S exhibited similar inactivation rates (quantified at +40 mV, fitted with a double exponential relationship; Figure 2C). For both Kv4.3L and Kv4.3S, co-expression with KChIP2b diminished the slow component of inactivation but slowed the fast component, such that current decay was well-described by a single exponential function. Strikingly, however, Kv4.3L-KChIP2b current inactivated 40% more slowly than Kv4.3S-KChIP2b current (Figure 2D). Steady-state inactivation was compared using a double-pulse protocol to quantify the fraction of available channels at +40 mV following prepulses to a range of voltages. Homomeric Kv4.3L and Kv4.3S currents were indistinguishable, whereas KChIP2b right-shifted the voltage dependence of Kv4.3L steady-state inactivation +5 mV more than for Kv4.3S. Thus, despite augmenting Kv4.3L current less efficiently, KChIP2b was more effective at shifting Kv4.3L inactivation voltage dependence, compared to that of Kv4.3S (Figure 2E). In contrast, recovery from inactivation, quantified by a double-pulse protocol of fixed-voltage pulses with variable interpulse intervals, was similar for Kv4.3L and Kv4.3S alone, and similarly speeded by KChIP2b for both isoforms (Figure 2F). Values for curve fits for all parameters and all subunit combinations tested are given in Table 1 (steady-state inactivation) and Table 2 (recovery from inactivation).

**Table 1:** **Steady-state inactivation parameters**.

**Subunits**	**V_0.5_ (mV)**	**Slope (1/mV)**	***R*****^2^**
Kv4.3L	−61.4 ± 0.34	6.9 ± 0.47	0.998
Kv4.3S	−61.4 ± 0.37	5.8 ± 0.27	0.9991
Kv4.3L-KChIP2b	−49.0 ± 0.78	4.4 ± 0.34	0.9999
Kv4.3S-KChIP2b	−54.0 ± 0.78	4.0 ± 0.36	0.9994
Kv4.3L-KCNE2	−60.6 ± 0.54	6.7 ± 0.49	0.999
Kv4.3L-KCNE3	−60.5 ± 061	6.4 ± 0.40	0.9992
Kv4.3L-KCNE5	−63.3 ± 0.82	7.1 ± 0.44	0.9998
Kv4.3S-KCNE2	−57.9 ± 0.87	6.6 ± 0.44	0.9995
Kv4.3S-KCNE3	−61.4 ± 0.52	5.9 ± 0.39	0.9985
Kv4.3S-KCNE5	−62.2 ± 0.52	6.6 ± 0.31	0.9984
Kv4.3L-KChIP2b-KCNE1	−53.1 ± 0.31	4.5 ± 0.16	0.9997
Kv4.3L-KChIP2b-KCNE2	−53.1 ± 0.2	4.3 ± 0.16	0.9998
Kv4.3L-KChIP2b-KCNE3	−56.4 ± 0.72	4.1 ± 0.3	0.9998
Kv4.3L-KChIP2b-KCNE4	−53.2 ± 0.42	4.5 ± 0.3	0.9998
Kv4.3L-KChIP2b-KCNE5	−49.5 ± 0.77	5.2 ± 0.4	0.9979
Kv4.3S-KChIP2b-KCNE1	−56.9 ± 0.36	4.0 ± 0.22	0.9999
Kv4.3S-KChIP2b-KCNE2	−59.6 ± 0.39	3.5 ± 0.16	0.9999
Kv4.3S-KChIP2b-KCNE3	−54.1 ± 0.27	3.8 ± 0.15	0.9999
Kv4.3S-KChIP2b-KCNE4	−52.9 ± 0.42	4.7 ± 0.2	0.9998
Kv4.3L-Kv4.3S-KChIP2b-KCNE4	−54.6 ± 0.82	3.9 ± 0.36	0.9999
Kv4.3S-KChIP2b-KCNE5	−53.6 ± 1.6	5.2 ± 0.64	0.9999

*Fits were made to mean data and therefore P-values are not available for comparisons between groups, but R^2^-values are given to indicate goodness of fit. Graphs and n-values are given in figures and figure legends*.

**Table 2:** **Inactivation recovery kinetics**.

**Subunits**	**τ_fast_ (ms)**	**A_fast_**	**τ_slow_ (ms)**	**A_slow_**	***R*****^2^**
Kv4.3L	275 ± 73	0.42 ± 0.13	1066 ± 263	0.45 ± 0.13	0.9999
Kv4.3S	298 ± 63	0.55 ± 0.14	1110 ± 366	0.37 ± 0.14	0.9999
Kv4.3L-KChIP2b	75 ± 4.4	1.07 ± 0.03	n/a	n/a	0.9993
Kv4.3S-KChIP2b	74 ± 4.8	1.07 ± 0.04	n/a	n/a	0.9994
Kv4.3L-KCNE1	100 ± 55	0.2 ± 0.1	718 ± 151	0.64 ± 0.09	0.9997
Kv4.3L-KCNE2	251 ± 17	0.43 ± 0.03	1038 ± 53	0.50 ± 0.03	0.9999
Kv4.3L-KCNE5	309 ± 21	0.83 ± 0.05	1962 ± 2873	0.06 ± 0.04	0.9996
Kv4.3S-KCNE1	n/a	n/a	610 ± 110	0.16 ± 0.01	0.998
Kv4.3S-KCNE2	322 ± 132	0.39 ± 0.45	616 ± 172	0.53 ± 0.46	0.9998
Kv4.3S-KCNE5	361 ± 17	0.72 ± 0.01	n/a	n/a	0.9984
Kv4.3L-KChIP2b-KCNE1	117 ± 6.8	1.04 ± 0.04	n/a	n/a	0.999
Kv4.3L-KChIP2b-KCNE2	103 ± 3.2	1.21 ± 0.03	n/a	n/a	0.9997
Kv4.3L-KChIP2b-KCNE3	103 ± 3.8	1.1 ± 0.03	n/a	n/a	0.9995
Kv4.3L-KChIP2b-KCNE4	84.8 ± 2.8	1.14 ± 0.03	n/a	n/a	0.9997
Kv4.3L-KChIP2b-KCNE5	73.0 ± 1.65	1.19 ± 0.02	n/a	n/a	0.9998
Kv4.3S-KChIP2b-KCNE1	63.7 ± 1.02	1.09 ± 0.01	n/a	n/a	0.9997
Kv4.3S-KChIP2b-KCNE2	106 ± 4.0	1.13 ± 0.02	n/a	n/a	0.9989
Kv4.3S-KChIP2b-KCNE3	73.6 ± 1.5	1.16 ± 0.02	n/a	n/a	0.9998
Kv4.3S-KChIP2b-KCNE4	55.8 ± 1.64	1.32 ± 0.05	n/a	n/a	0.9997
Kv4.3L-Kv4.3S-KChIP2b-KCNE4	59.85 ± 0.78	1.12 ± 0.01	n/a	n/a	0.9995
Kv4.3S-KChIP2b-KCNE5	67.4 ± 0.88	1.08 ± 0.01	n/a	n/a	0.9998

*Fits were made to mean data and therefore P-values are not available for comparisons between groups, but R^2^-values are given to indicate goodness of fit. Graphs and n-values are given in figures and figure legends; n/a, not applicable (single exponential fit)*.

### KCNE1 differentially regulates inactivation of Kv4.3S vs. Kv4.3L

**Figure 3:**
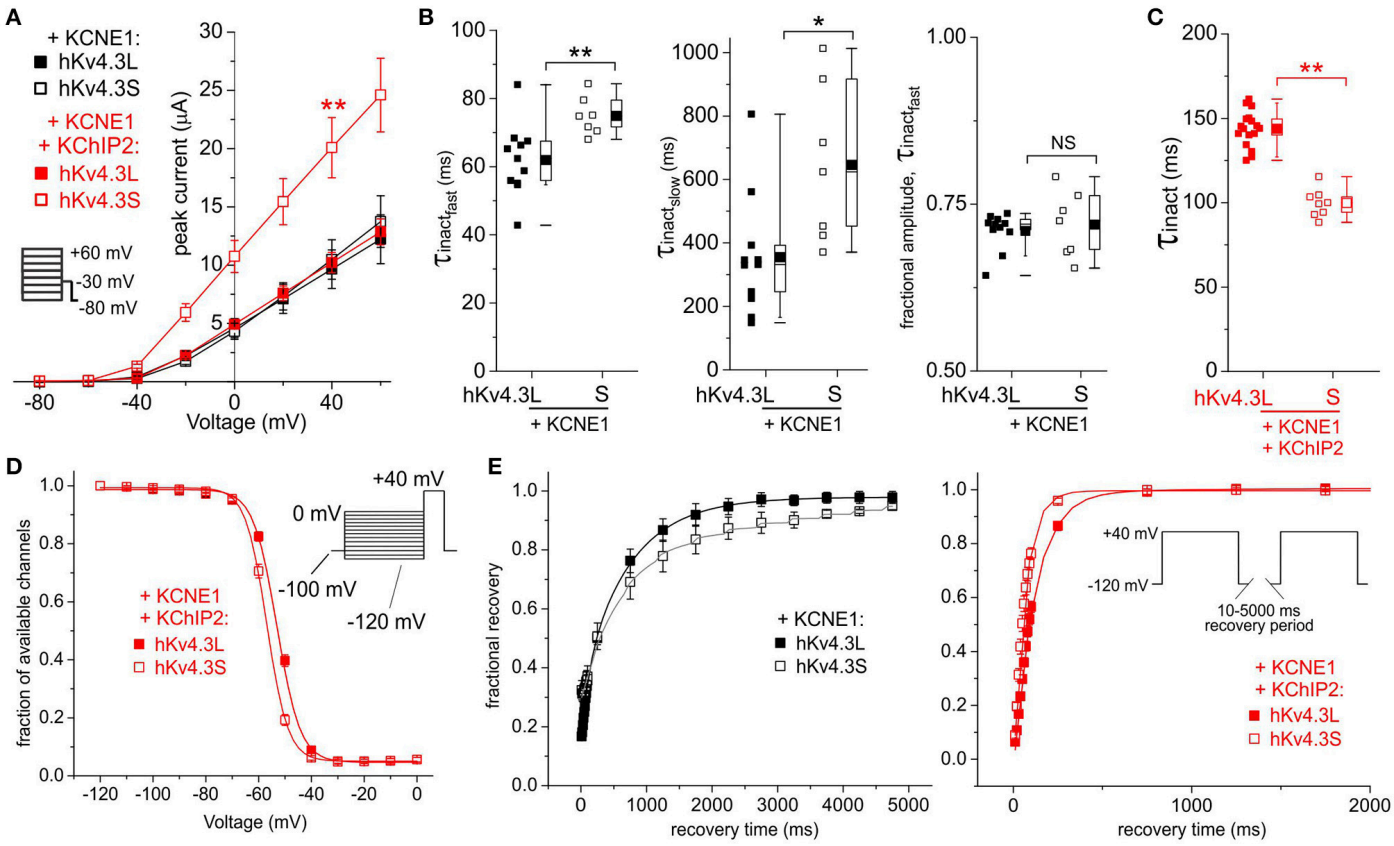
**KCNE1 differentially affects Kv4.3L and Kv4.3S function with/without KChIP2b. (A)** Mean ± SEM peak current/voltage relationship for current traces recorded from *Xenopus* oocytes 48 h after injection of cRNA encoding Kv4.3L or Kv4.3S (1 ng) with KCNE1 (5 ng), with or without KChIP2b (5 ng; *n* = 9–17). *Inset*: Voltage clamp protocol. ^**^P < 0.01 compared to other currents at +40 mV. **(B)** Box plots showing individual and mean ± SEM values for τ of fast and slow components, and relative amplitude, of fast inactivation (double exponential fit) of Kv4.3L-KCNE1 vs. Kv4.3S-KCNE1 currents recorded as in **(A)**; *n* = 7–11. ^*^P < 0.05; ^**^P < 0.01; NS, *P* > 0.05. **(C)** Box plot showing individual and mean ± SEM values for τ of Kv4.3L-KCNE1-KChIP2b vs. Kv4.3S-KCNE1-KChIP2b inactivation (single exponential fit) for currents recorded as in **(B)**; *n* = 8–17. ^**^P < 0.01. **(D)** Mean ± SEM fraction of available channels/voltage relationship for Kv4.3L-KCNE1-KChIP2b vs. Kv4.3S-KCNE1-KChIP2b currents; voltage protocol upper right inset; *n* = 6. **(E)** Mean ± SEM fractional recovery from inactivation/recovery time for Kv4.3L-KCNE1 vs. Kv4.3S-KCNE1 with (*n* = 6) or without (*n* = 3–4) KChIP2b; voltage protocol upper right inset.

In the absence of KChIP2b, co-expression of KCNE1 did not differentially affect Kv4.3L vs. Kv4.3S current magnitude; in contrast, when co-expressed with both KChIP2b and KCNE1, Kv4.3S current was twice that of Kv4.3L (Figure 3A) as observed in the absence of KCNE1 (Figures 2A,B). However, KCNE1 slowed Kv4.3S inactivation more than that of Kv4.3L (Figure 3B). Kv4.3L-KCNE1-KChIP2b was >40% slower inactivating than Kv4.3S-KCNE1-KChIP2b (Figure 3C), similar to effects in the absence of KCNE1 (Figure 2D). Steady-state inactivation in the absence of KChIP2b could not be compared because of contamination in this longer protocol by activation of endogenous oocyte KCNQ1 by KCNE1, but in the presence of KChIP2 these currents did not noticeably overlap with those of Kv4.3, which showed a larger positive shift in steady-state inactivation for Kv4.3L than Kv4.3S, with KCNE1 and KChIP2b (Figure 3D), as also observed without KCNE1 (Figure 2E). Recovery from inactivation was slower for Kv4.3S than Kv4.3L channels co-expressed with KCNE1, but this effect was reversed by KChIP2b, with recovery being twice as fast for channels containing Kv4.3S compared to Kv4.3L (Figure 3E).

### KCNE2 preferentially speeds inactivation recovery of Kv4.3S vs. Kv4.3L

**Figure 4:**
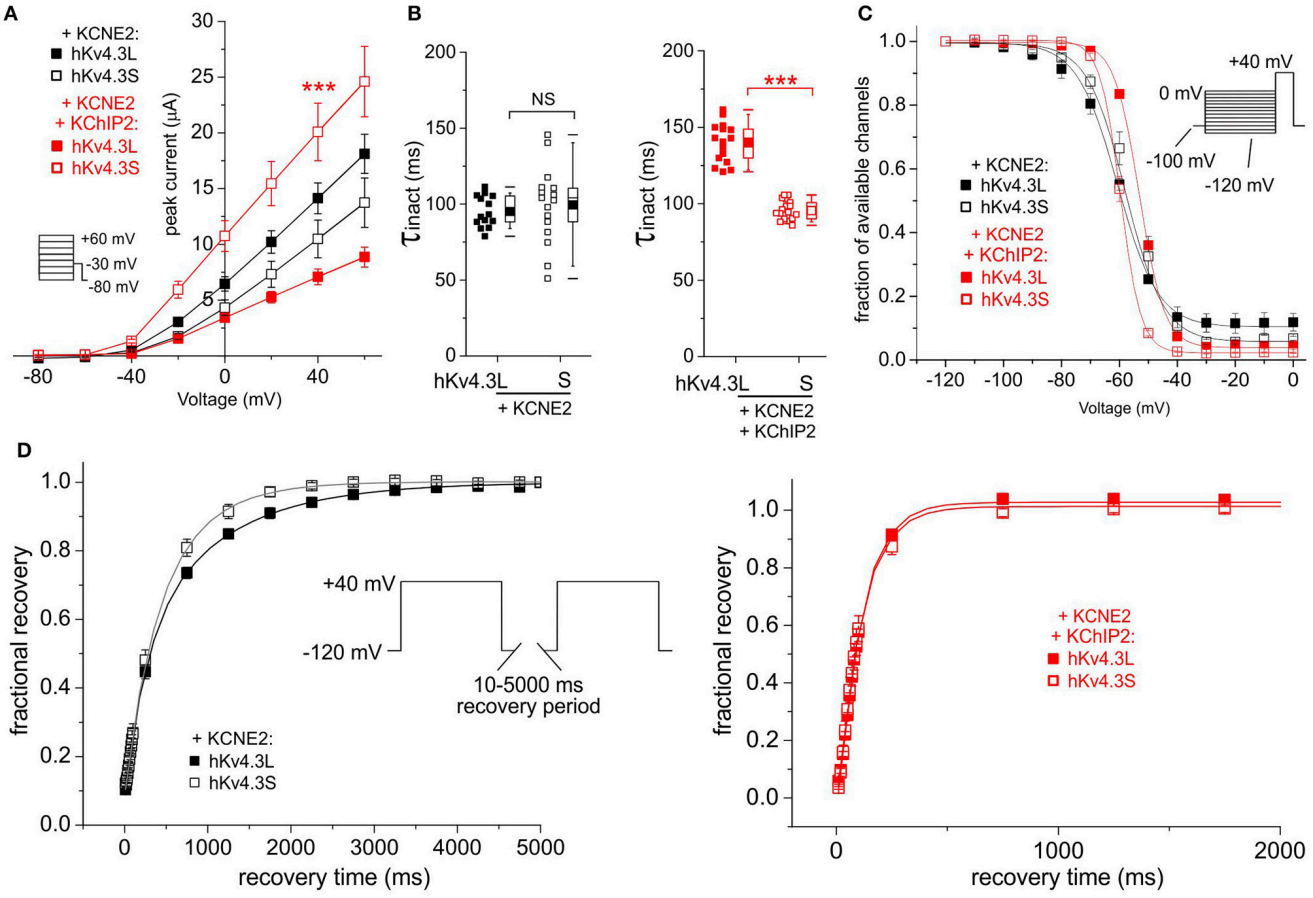
**KCNE2 differentially affects Kv4.3L and Kv4.3S function with/without KChIP2b. (A)** Mean ± SEM peak current/voltage relationship for current traces recorded from *Xenopus* oocytes 36–48 h after injection of cRNA encoding Kv4.3L or Kv4.3S (1 ng) with KCNE2 (5 ng), with or without KChIP2 (5 ng; *n* = 14–18). *Inset*: Voltage clamp protocol. ^***^P < 0.001 compared to Kv4.3L-KChIP2-KCNE2 at +40 mV. **(B)** Box plot showing individual and mean ± SEM values for τ of inactivation (single exponential fit) for currents recorded as in **(A)**; *n* = 14–18. NS, *P* > 0.05; ^***^P < 5 × 10^−14^. **(C)** Mean ± SEM fraction of available channels/voltage relationship for Kv4.3L-KCNE2 vs. Kv4.3S-KCNE2 (upper) and Kv4.3L-KCNE2-KChIP2 vs. Kv4.3S-KCNE2-KChIP2 (lower) currents; voltage protocol upper right inset; *n* = 6. **(D)** Mean ± SEM fractional recovery from inactivation/recovery time for Kv4.3L-KCNE2 vs. Kv4.3S-KCNE2 (upper) and Kv4.3L-KCNE2-KChIP2 vs. Kv4.3S-KCNE2-KChIP2 (lower) currents; voltage protocol upper inset; *n* = 5–7.

In the absence of KChIP2b, KCNE2 did not differentially affect Kv4.3L vs. Kv4.3S current magnitude; with KChIP2b and KCNE2, Kv4.3S current was almost 3-fold that of Kv4.3L (Figure 4A). KCNE2 similarly converted Kv4.3L and Kv4.3S inactivation to a single-exponential decay without differentially altering the rates, but co-expression with KChIP2b left Kv4.3S-KCNE2 inactivation unaffected, while slowing that of Kv4.3l-KCNE2 by 40% (Figure 4B). Steady-state inactivation of Kv4.3 was isoform-independent with KCNE2, but co-expression of KChIP2 again shifted voltage dependence by 5 mV more in tripartite channels formed with Kv4.3L (Figure 4C). KCNE2 preferentially speeded recovery from inactivation of Kv4.3S vs. Kv4.3L, but only in the absence of KChIP2b (Figure 4D).

### KCNE3 alleviates Kv4.3 isoform-dependence of KChIP2b effects on inactivation

**Figure 5:**
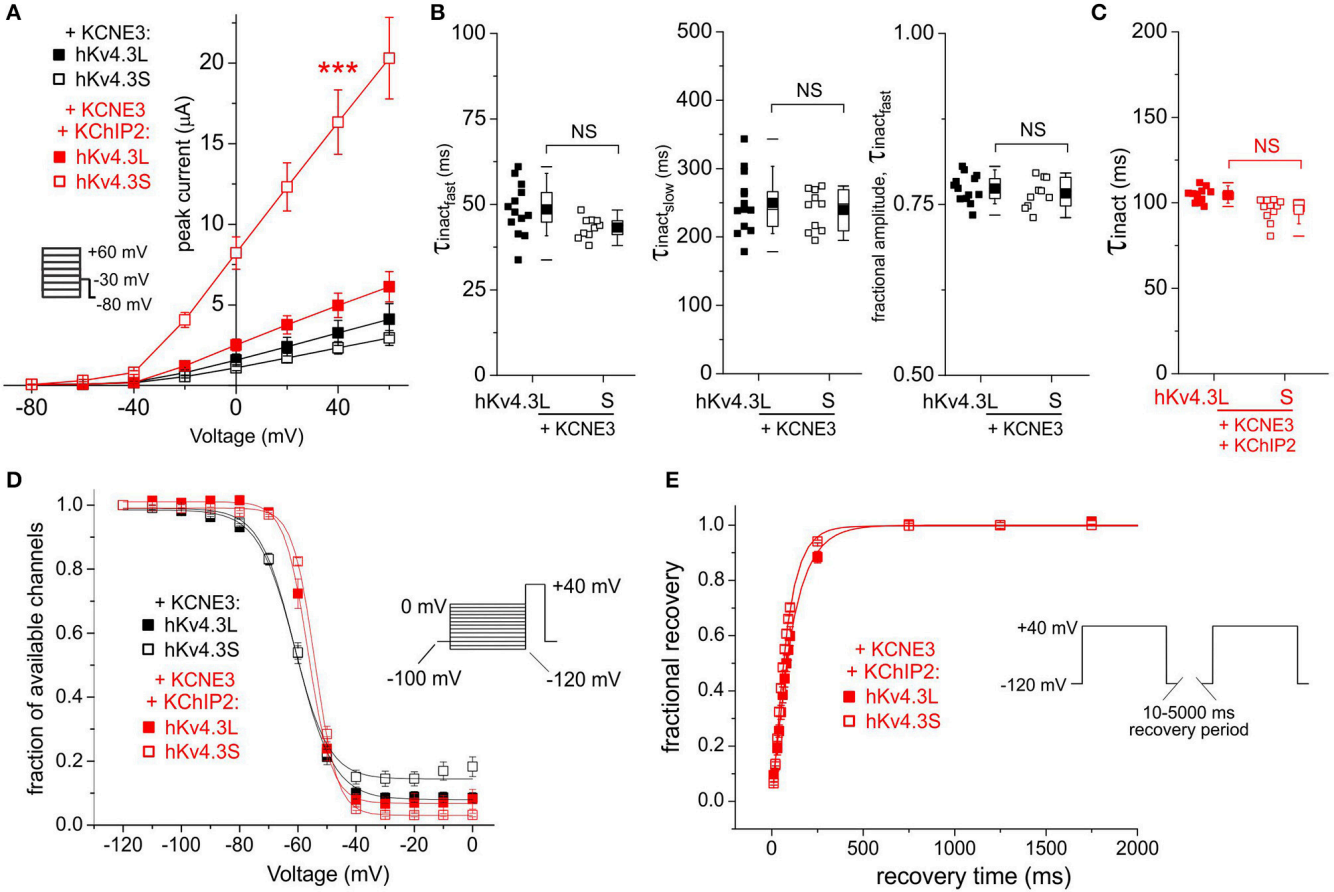
**KCNE3 differentially affects Kv4.3L and Kv4.3S current magnitude with KChIP2b. (A)** Mean ± SEM peak current/voltage relationship for current traces recorded from *Xenopus* oocytes 36–48 h after injection of cRNA encoding Kv4.3L or Kv4.3S (1 ng) with KCNE3 (5 ng) with or without KChIP2 (5 ng; *n* = 11–20). *Inset*: Voltage clamp protocol. ^***^P < 0.001 compared to other currents at +40 mV. **(B)** Box plots showing individual and mean ± SEM values for τ of fast and slow components, and relative amplitude, of fast inactivation (double exponential fit) of Kv4.3L-KCNE3 vs. Kv4.3S-KCNE3 currents recorded as in **(A)**; *n* = 10–13. NS, *P* > 0.05. **(C)** Box plot showing individual and mean ± SEM values for τ of Kv4.3L-KCNE3-KChIP2 vs. Kv4.3S-KCNE3-KChIP2 inactivation (single exponential fit) for currents recorded as in **(B)**; *n* = 11–15. NS, *P* > 0.05. **(D)** Mean ± SEM fraction of available channels/voltage relationship for Kv4.3L-KCNE3 vs. Kv4.3S-KCNE3 with/without KChIP2; voltage protocol upper right inset; *n* = 5–7. **(E)** Mean ± SEM fractional recovery from inactivation/recovery time for Kv4.3L-KCNE3-KChIP2 vs. Kv4.3S-KCNE3-KChIP2; voltage protocol upper inset; *n* = 4–7.

KCNE3 was equally inhibitory to Kv4.3L and Kv4.3S, an effect alleviated by KChIP2b only for Kv4.3S (increasing current to 7-fold that in the absence of KChIP2b; Figure 5A). Regardless of KChIP2b expression, Kv4.3 inactivation rate and voltage dependence were isoform-independent when co-expressed with KCNE3 (Figures 5B–D). KCNE3 appeared to marginally accelerate Kv4.3S-KChIP2 recovery from inactivation compared to Kv4.3L-KChIP2, but this apparent difference possibly arose from the large mismatch in current magnitude between the two subunit combinations (Figure 5E). Low current magnitudes prevented accurate quantification of recovery in the absence of KChIP2b.

### KCNE4 Kv4.3-isoform-dependently inhibits Kv4.3-KChIP2b and accelerates its inactivation and recovery

**Figure 6:**
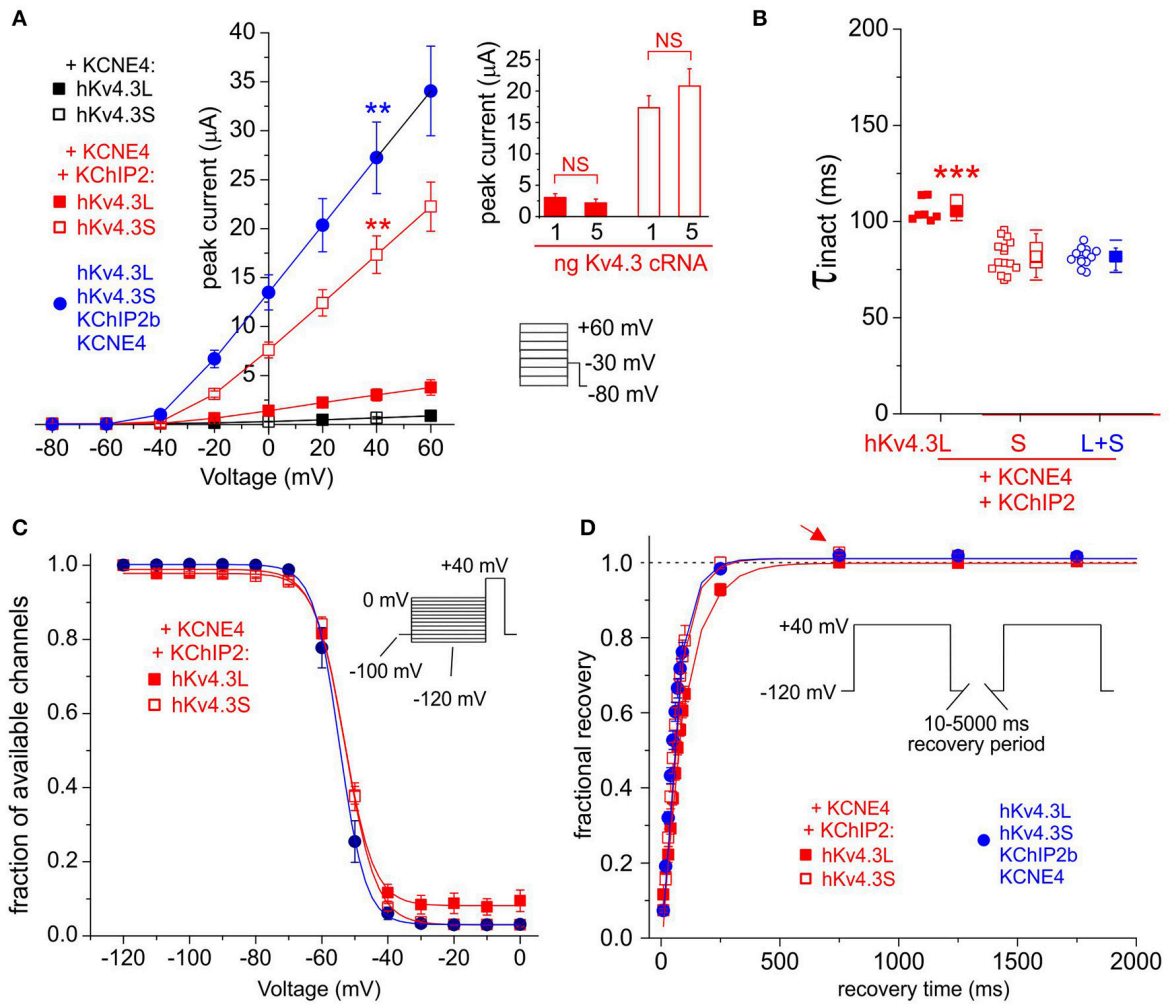
**KCNE4 differentially affects Kv4.3L and Kv4.3S function with KChIP2b. (A)** Mean ± SEM peak current/voltage relationship for current traces recorded from *Xenopus* oocytes 36–48 h after injection of cRNA encoding Kv4.3L (filled squares) and (circles)/or (open squares) Kv4.3S (1 ng), with KCNE4 (5 ng), with/without KChIP2 (5 ng; *n* = 10–17). *Lower right inset*: Voltage clamp protocol. ^**^P < 0.01 compared to all other currents at +40 mV, by one-way ANOVA with *post-hoc* Tukey HSD-test. *Upper right inset:* no effect on current density of individually increasing Kv4.3L (red columns) or Kv4.3S (open columns) cRNA injected, from 1 to 5 ng per oocyte, when co-injected with KCNE4S and KChIP2b cRNA (*n* = 11–17). NS = *P* > 0.05 by one-way ANOVA with *post-hoc* Tukey HSD-test; all other group comparisons *P* < 0.01. **(B)** Box plot showing individual and mean ± SEM values for τ of inactivation (single exponential fit) for Kv4.3L and / or Kv4.3S co-expressed with KCNE4 and KChIP2; *n* = 7–15. ^***^P < 5 × 10^−6^ vs. other groups; other comparisons *P* > 0.05. **(C)** Mean ± SEM fraction of available channels/voltage relationship for Kv4.3L and/or Kv4.3S co-expressed with KCNE4 and KChIP2; voltage protocol upper right inset; *n* = 5–6. **(D)** Mean ± SEM fractional recovery from inactivation/recovery time for Kv4.3L and/or Kv4.3S co-expressed with KCNE4 and KChIP2; voltage protocol upper inset; *n* = 5–6. Arrow, overshoot following recovery from inactivation.

KCNE4 potently and similarly inhibited Kv4.3L and Kv4.3S, while KChIP2b strikingly amplified Kv4.3S-KCNE4 current 25-fold, compared to only 5-fold for Kv4.3L-KCNE4 (Figure 6A). Co-injection of 1 + 1 ng Kv4.3L and Kv4.3S with KChIP2b and KCNE4 (5 + 5 ng) further enhanced peak current, to ~40-fold that of Kv4.3L or Kv4.3S with KCNE4 in the absence of KChIP2b (Figure 6A). This was not simply a summation arising from injecting 2 ng total Kv4.3 cRNA per oocyte, because increasing cRNA of either Kv4.3 isoform alone to 5 ng did not increase current at +40 mV beyond that of 1 ng of single Kv4.3 isoform-injected oocytes, when co-expressed with KCNE4 and KChIP2b (Figure 6A *inset*). Analysis of Kv4.3-KCNE4 inactivation was hampered by low current magnitude, but facilitated by KChIP2b co-expression. KCNE4 accelerated Kv4.3L-KChIP2 and Kv4.3S-KChIP2b inactivation (Figure 6B) by 20–30% compared to in the absence of KCNE4 (Figure 2D), preserving the Kv4.3 isoform-dependence, i.e., faster inactivation of Kv4.3S-KChIP2 compared to Kv4.3L-KChIP2 (Figure 6B). While Kv4.3 steady-state inactivation was isoform-independent when co-expressed with KCNE4 and KChIP2b (Figure 6C), Kv4.3S-based channels recovered from inactivation faster than Kv4.3L-based complexes and the former also exhibited overshoot whereas the latter did not (Figure 6D). Interestingly, channels generated by expression of both Kv4.3L and Kv4.3S together with KChIP2b and KCNE4 (circles) had similar rates of inactivation and recovery from inactivation to Kv4.3S-KChIP2b-KCNE4 but not Kv4.3L-KChIP2b-KCNE4 (Figures 6B,D) while steady-state inactivation was similar for all three groups (Figure 6C).

### KCNE5 inhibits Kv4.3S more than Kv4.3L

**Figure 7:**
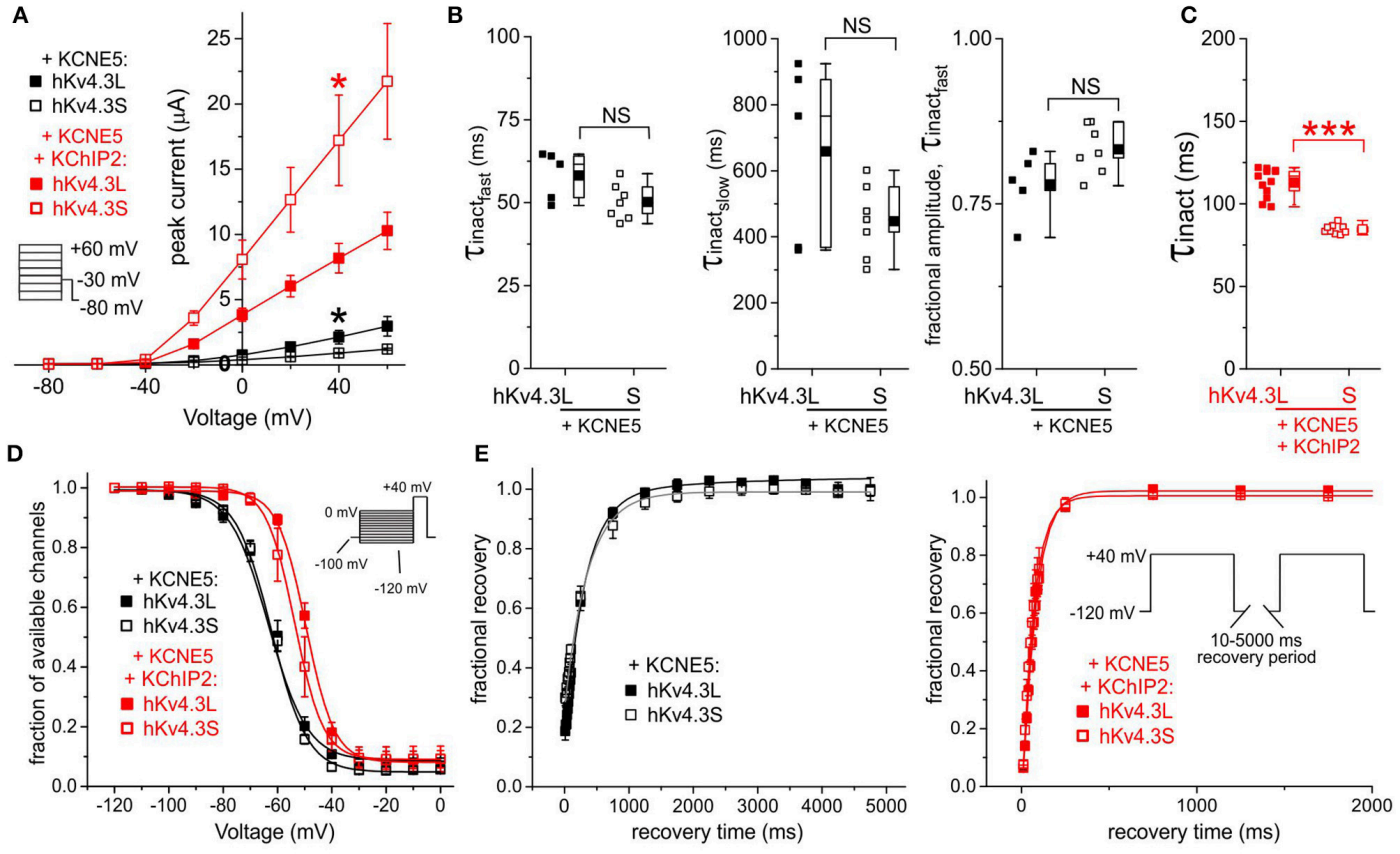
**KCNE5 differentially affects Kv4.3L and Kv4.3S function. (A)** Mean ± SEM peak current/voltage relationship for current traces recorded from *Xenopus* oocytes 36–48 h after injection of cRNA encoding Kv4.3L or Kv4.3S (1 ng) with KCNE5 (1–5 ng) with/without KChIP2b (5 ng; *n* = 6–12). ^*^P < 0.05 between Kv4.3L and Kv4.3S with similar β subunits. *Inset*: Voltage clamp protocol. **(B)** Box plots showing individual and mean ± SEM values for τ of fast and slow components, and relative amplitude, of fast inactivation (double exponential fit) of Kv4.3L-KCNE5 vs. Kv4.3S-KCNE5 currents recorded as in **(A)**; *n* = 5–7. NS, *P* > 0.05. **(C)** Box plot showing individual and mean ± SEM values for τ of inactivation (single exponential fit) for Kv4.3L vs. Kv4.3S co-expressed with KCNE5 and KChIP2; *n* = 9–12. ^***^P < 5 × 10^−8^. **(D)** Mean ± SEM fraction of available channels/voltage relationship for Kv4.3L-KCNE5 vs. Kv4.3S-KCNE5 channels with (*n* = 5–12) or without (*n* = 5–9) KChIP2; voltage protocol inset. **(E)** Mean ± SEM fractional recovery from inactivation/recovery time for Kv4.3L-KCNE5 vs. Kv4.3S-KCNE5 channels with (*n* = 7–8) or without (*n* = 5–6) KChIP2; voltage protocol inset.

KCNE5 inhibited Kv4.3S activity such that it was ~3-fold lower than that of Kv4.3L; KChIP2b effectively amplified Kv4.3S-KCNE5 current to a level similar to that in the absence of KCNE5 but only restored Kv4.3L current to half this amount, similar to that of Kv4.3L alone (Figures 2A,B, 7A). Kv4.3-KCNE5 inactivation rate, voltage dependence and recovery were isoform-independent without KChIP2b. With KChIP2b and KCNE5 co-expression, there were two isoform-dependent differences: Kv4.3S inactivation was again faster than that of Kv4.3L, whereas the steady-state inactivation of the latter had a 4 mV more positive V_0.5_ compared to Kv4.3S (Figures 7C–E).

### Kv4.3S-KChIP2 function is modified by a peptide corresponding to Kv4.3L residues 488–506

**Figure 8:**
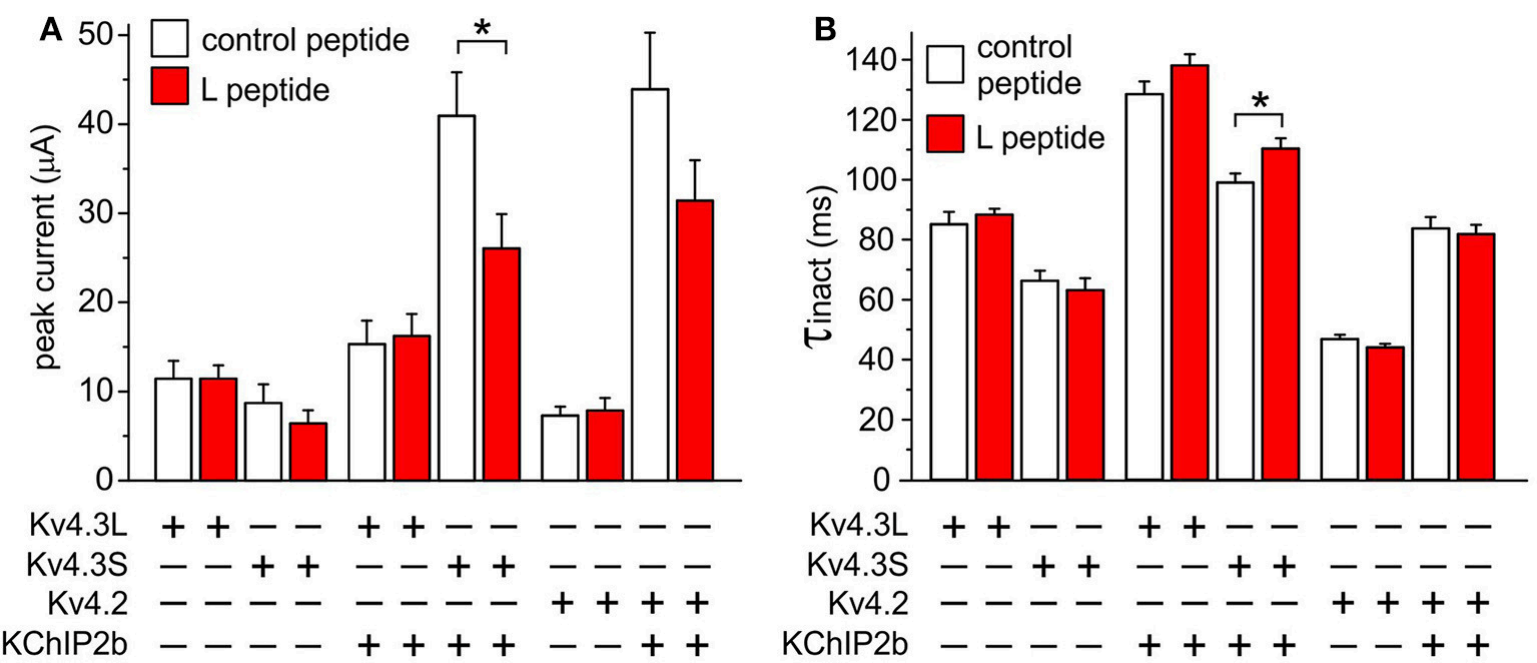
**Kv4.3S-KChIP2 function is modified by a peptide corresponding to Kv4.3L residues 488–506. (A)** Mean ± SEM peak current at 40 mV for current traces recorded from *Xenopus* oocytes 48–72 h after injection of 1.5 ng cRNA each encoding the following: Kv4.2 with (*n* = 10) or without (*n* = 7–8) KChIP2; Kv4.3L with (*n* = 13–20) or without (*n* = 8–12) KChIP2; Kv4.3S with (*n* = 13–17) or without (*n* = 8) KChIP2, 30–90 min following injection of L peptide (red columns) or control peptide (open columns; final concentration 25 μM). ^*^P < 0.05; all other same-subunit combination comparisons *P* > 0.05. **(B)** Mean ± SEM inactivation τ at 40 mV for current traces recorded from *Xenopus* oocytes 48–72 h after injection of cRNAs as in **(A)**. ^*^P < 0.05; all other same-subunit combination comparisons *P* > 0.05.

Functional differences between Kv4.3S and Kv4.3L in the presence of KChIP2b were partially alleviated by injection, 30–90 min prior to TEVC recording, of a peptide (25 μM) corresponding to the Kv4.3L-specific C-terminal 19-residue stretch, residues 488–506 (“L peptide”). As a control, currents were compared to those of oocytes injected with a sequence-scrambled 19-mer control peptide. The L peptide reduced Kv4.3S-KChIP2 currents by 36% (*P* = 0.02, *n* = 13–17) but had no effects on current magnitude of Kv4.3S in the absence of KChIP2b, nor on Kv4.3L in the absence or presence of KChIP2b (Figure 8A). The L peptide did not alter homomeric Kv4.2 current magnitude, but reduced Kv4.2-KChIP2b current magnitude by 28.5%, although this did not reach statistical significance (*P* = 0.13, *n* = 12–13). In addition, the L peptide slowed Kv4.3S-KChIP2b inactivation rate 11% (*P* = 0.03, *n* = 12–17) but had no statistically significant effects on the inactivation rate of channels formed from other subunit combinations (Figure 8B). Note that for simplicity of comparisons, for peptide experiments inactivation rates were fitted with a single exponential regardless of KChIP2 presence or absence.

### Kv4.3S- and KChIP2-dependent current reduction with subunit ratios mimicking those in human HF

**Figure 9:**
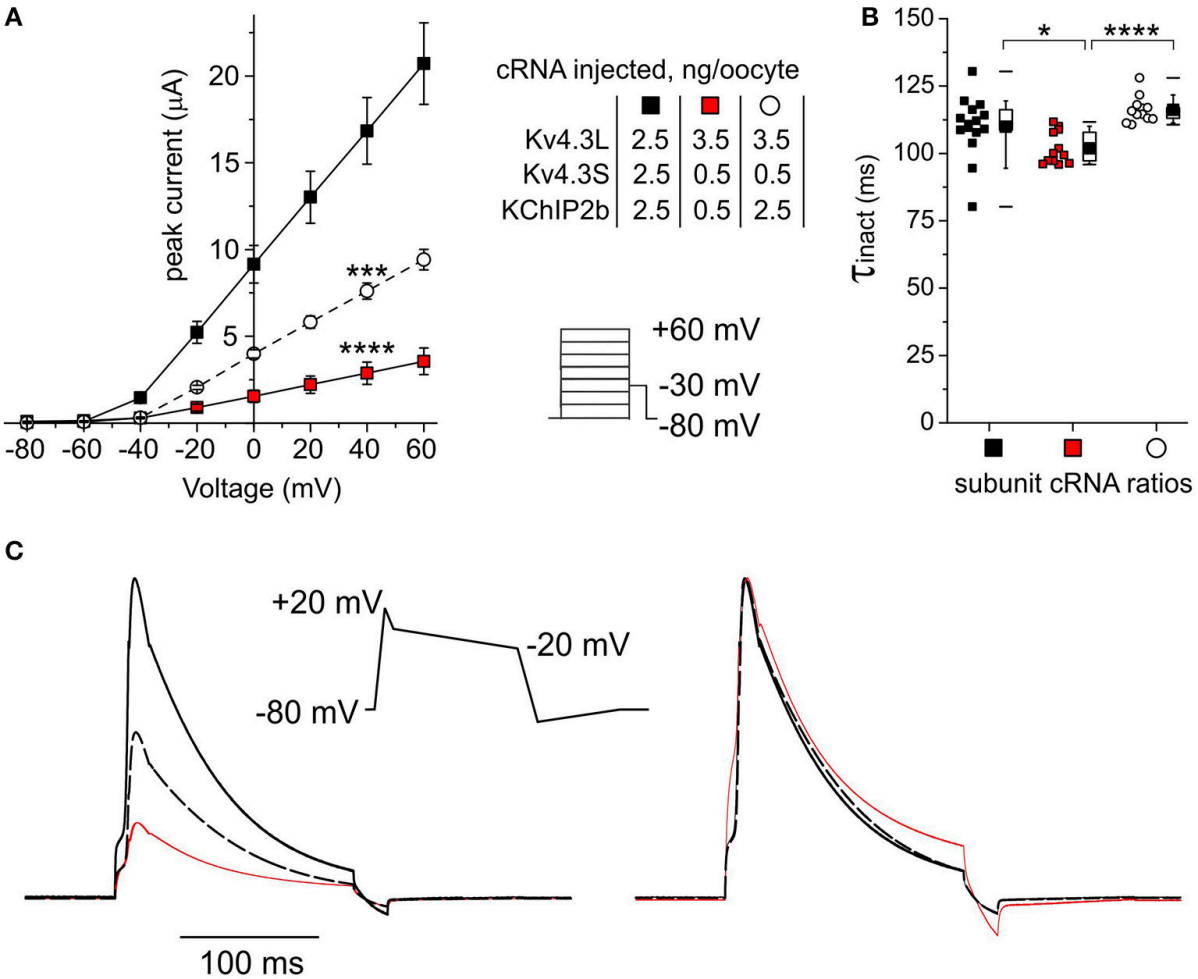
**Subunit cRNA ratios mimicking those in human HF Kv4.3S- and KChIP2-dependently reduce current. (A)** Mean ± SEM peak current/Voltage relationship for current traces recorded from *Xenopus* oocytes 48–72 h after injection of cRNAs as indicated (*n* = 12–15; ^***^P < 0.001, ^****^P < 1 × 10^−5^ vs. current magnitude for other groups at +40 mV). *Lower right inset*: Voltage clamp protocol. **(B)** Box plot showing individual and mean ± SEM values for τ of inactivation (single exponential fit) for currents generated as in **(A)**, symbols as in **(A)** (*n* = 12–15; ^*^P < 0.05, ^****^P < 1 × 10^−5^). **(C)** Averaged current traces elicited by ventricular action potential-like Voltage protocol (center) recorded in oocytes expressing channel subunit ratios as in **(A)**, line colors as in **(A)**, scaled to mean current magnitudes as in **(A)** (left) or normalized for easier comparison of gating kinetics (right). Each trace is an average of 120–300 raw traces recorded from 4 to 10 cells.

Lastly, Kv4.3L, Kv4.3S, and KChIP2 cRNAs were injected into oocytes in ratios corresponding to these previously reported for normal vs. failing human hearts (Radicke et al., 2006), and current properties examined. Compared to oocytes injected with equal amounts (2.5 ng of each) of all three cRNAs (to approximate the conditions reported for non-failing hearts), injection instead of 3.5 ng Kv4.3L with 0.5 ng each of Kv4.3S and KChIP2b (approximating the ratio in HF) reduced peak current 6-fold at +40 mV (*P* = 1.5 × 10^−6^; *n* = 12–17). Injection of 3.5 ng Kv4.3L with 0.5 ng of Kv4.3S to mimic reduction in the latter observed in HF, but with a “normal” amount of KChIP2b (2.5 ng), generated an intermediate current level (Figure 9A). Thus, reductions in either Kv4.3S or KChIP2b approximating conditions in HF are sufficient to diminish total Kv4.3 current levels. Compared to currents generated by injection of equal amounts of cRNA for Kv4.3L, Kv4.3S and KChIP2b (2.5 + 2.5 + 2.5 ng), the HF-mimicking subunit cRNA ratio (3.5 + 0.5 + 0.5 ng) speeded total Kv4.3 inactivation by 9% (*P* < 0.05, *n* = 12–15) whereas reducing solely the Kv4.3S amount had no effect (Figure 9B).

Currents elicited by Voltage clamp protocols mimicking the morphology of a generic human ventricular action potential showed negligible differences in the kinetics of responses to each Voltage ramp (Figure 9C). Pulsing of oocytes at a frequency of 1 Hz using this protocol did not result in diminishment of current magnitude for any of the subunit combinations tested, showing that inactivation recovery was not compromised in a meaningful manner by any of the subunit expression changes; this result also held in additional studies in which the depolarizing pulse duration and/or the pulse frequency was doubled (data not shown). As the oocyte studies are conducted at room temperature, these rates more than cover the gating kinetics that would be required of *I*_to_ channels at body temperature *in vivo*. Thus, the paramount *I*_to_ manifestation (and source of any associated pathology) of the HF-associated changes in subunit expression *in vivo* would be predicted to be the considerable drop in current magnitude in response to rapid depolarization (Figure 9A), rather than changes in Voltage-dependence or gating kinetics.

## Discussion

Native fast-inactivating Kv currents are generated predominantly by channels containing Kv1.4, Kv3.3, Kv3.4, or Kv4.x α subunits, all of which exhibit inherent fast-inactivation apparatus [N-type inactivation domains, although the mechanistic nuances are different for Kv4.x α subunits (Bärghaan et al., 2008; Dougherty et al., 2008; Barghaan and Bähring, 2009)]. In the case of Kv1 and Kv3 subfamily channels, heteromers of α subunits containing both N-type (fast-inactivating) and delayed rectifier (slow, C-type inactivating) α subunits can generate currents with intermediate inactivation kinetics, proportional to the number of inactivation domains available to plug the pore after channel opening. Regulatory subunits, including protein kinases and KCNEs, may further modify Kv1 and Kv3 channel inactivation rates, and other factors including voltage dependence, surface expression, and even α subunit composition itself (Abbott et al., 2001, 2006; Grunnet et al., 2003; Kanda et al., 2011a,b). Kv4 channels are different in that there are no delayed rectifier α subunits in the Kv4 subfamily, so the channels they form are always fast-inactivating. Kv4 channels are, however, subject to modulation by several classes of regulatory proteins, thought to occur concurrently *in vivo* in human cardiac and neuronal Kv4-based macromolecular complexes. The major new finding in the present study is that beta subunits unlock previously unrecognized functional differences in Kv4.3 splice variants known to be expressed in human heart. This is of mechanistic importance, and is also potentially relevant to cardiovascular disease, as it predicts that previously recognized, HF-associated changes in *I*_to_ subunit expression could have a robust functional impact, reducing I_to_ density by as much as 50%.

The prior assumption with respect to Kv4.3L and Kv4.3S splice variants was that their function differed only with respect to their regulation by PKC, via threonine 504 within the C-terminal 19-residue stretch (Po et al., 2001; Xie et al., 2009). Radicke and colleagues analyzed the functional effects of KChIP2 and KCNE subunits on Kv4.3L and also discovered HF-associated remodeling of Kv4.3 splicing and β subunits, but did not examine functional effects on Kv4.3S (Radicke et al., 2006). While Kv4.3L transcript was increased by 33% in HF, Kv4.3S transcript was decreased by 75%, resulting in a net reduction in total Kv4.3 transcript. KCNE4 was the highest-expressed KCNE subunit (128 fg/ng in normal heart; 75 fg/ng in HF) followed by KCNE2 (6.5 fg/ng in normal heart; 5.3 ng in HF). KCNE1 transcript was increased from 1.7 to 4.7 fg/ng in HF (Radicke et al., 2006).

Placed into the context of the prior findings for HF-associated subunit remodeling, the combination of subunit expression changes observed in the present study would be predicted to have complex but predominantly inhibitory effects on *I*_to_. KCNE4 (the long variant) was shown here to strongly inhibit Kv4.3L-KChIP2b current but have much weaker inhibitory effects on Kv4.3S-KChIP2b. Although KCNE4 expression dropped ~40% in human heart failure, it was still the predominant KCNE subunit. Therefore, if expressed in the same myocytes as Kv4.3 and KChIP2b, KCNE4 would be more inhibitory if the balance of Kv4.3 isoforms shifted heavily toward Kv4.3L as previously observed (Radicke et al., 2006). This effect would be even greater if normal hearts express heteromeric Kv4.3L-Kv4.3S-KChIP2b-KCNE4, as these generate even larger currents than Kv4.3S-KChIP2b-KCNE4, and would be predicted to be replaced by the lesser current-passing Kv4.3L-KChIP2b-KCNE4 (Figure 6). This inhibitory effect would be further exacerbated by reduced KChIP2 expression in failing hearts, as KChIP2b protects Kv4.3L from strong inhibition by KCNE4. Reduced KCNE5 in failing hearts would be predicted to increase *I*_to_, but this would be at least partially mitigated by the relatively stronger inhibitory effects of KCNE5 on Kv4.3L-KChIP2b channels compared to Kv4.3S-KChIP2b (Figure 7). Finally, KCNE1 upregulation in failing heart would be predicted to increase *I*_Ks_ [formed by KCNQ1 and KCNE1 (Barhanin et al., 1996; Sanguinetti et al., 1996)] and *I*_Kr_ [formed by hERG and one or more KCNE subunits, including KCNE1—which augments hERG current (McDonald et al., 1997)]. However, in the case of Kv4.3, we found that KCNE1 only augments the activity of channels comprising Kv4.3S and KChIP2b (Figure 3), so this would again mitigate augmentation of *I*_to_ by increased KCNE1.

Radicke and colleagues did not compare the effects of KCNE subunits on Kv4.3L in the absence of KChIP2, because in CHO cells they did not observe Kv4.3L currents in the absence of KChIP2 (Radicke et al., 2006). Nor has anyone reported comparing effects of β subunits on Kv4.3S vs. Kv4.3L, at least partly because the conserved Kv4 N-terminal was the acknowledged binding site for KChIPs (Bähring et al., 2001; Wang, 2008), whereas the Kv4.3L and Kv4.3S isoforms differ only in their C-termini. However, the C-terminal of closely-related α subunit Kv4.2 also mediates functional effects of KChIPs (Callsen et al., 2005; Han et al., 2006). The current work recapitulates this for Kv4.3 and refines it to the extent that a biologically and pathophysiologically relevant 19-residue segment in the Kv4.3 membrane-proximal C-terminal is shown to markedly influence the functional consequences of Kv4.3 co-assembly with two classes of β subunit.

Only select consequences of KChIP2b regulation of Kv4.3 function were attenuated by the 19-residue segment (current augmentation and slowing of inactivation), and others were augmented (larger shift in steady-state inactivation voltage dependence) or unaffected (equally robust increase in rate of inactivation recovery; similar shift from double- to single-exponential current decay kinetics during inactivation). Thus, the 19 residue segment is not suggested to interfere with Kv4.3-KChIP2b complex formation, instead selectively modulating functional consequences of the interaction. The finding here that a synthetic “L” peptide mimicking the 19-residue segment can acutely alter the effects of KChIP2 on Kv4.3S, altering current magnitude and inactivation kinetics to levels intermediate between those of Kv4.3S and Kv4.3L (Figure 8), strongly supports the premise that the 19-residue segment is directly involved in modulating some of the functional effects of KChIP2 on Kv4.3L. There may also be a reduction of the current augmenting effects of KChIP2b upon Kv4.2, which also lacks the 19-residue stretch unique to Kv4.3L, although this did not reach statistical significance. Effects of the L peptide on the function of Kv4.3S (and on Kv4.2) in complexes with KChIP2b are most likely to be via direct interaction with KChIP2, because the L peptide had no effects on channels in the absence of KChIP2. However, it is technically also feasible that presence of the L peptide, or the corresponding segment in the Kv4.3L α subunit, alters conformation of another domain within Kv4.3 in such a way that the α subunit responds differently to KChIP2b. Because effects were observed within 30 min of peptide injection, an alternative hypothesis, that the 19-residue segment only exerts influence on Kv4.3-KChIP2 complexes early in biogenesis, or dictates whether they co-assemble in the first place, is highly unlikely given that the turnover time of Kv4.3-KChIP2 complexes is much longer [e.g., *t*_1/2_ of decay in pulse chase studies of 7–9 h (Jiang et al., 2009)].

Results described above, together with the observation here that cRNA ratios approximating those previously observed in human HF (Radicke et al., 2006) dramatically reduce total Kv4.3 current (Figure 9), strongly suggests that the relative balance of Kv4.3L and Kv4.3S is crucial to dictating *I*_to_ density and kinetics when co-assembled with β subunits known to regulate Kv4.3 in human heart, and raises the idea that therapeutic targeting to specifically upregulate Kv4.3S would be more beneficial in terms of *I*_to_ density restoration in HF than blanket Kv4.3 upregulation.

It is important to also recognize limitations of the current study. *Xenopus* oocytes were used for channel expression, partly because their robust nature ensures a large quantity of recordings can be completed in a short time, facilitating direct comparison of many different subunit combinations within the same timeframe and same batch of oocytes. In addition, because *Xenopus* oocytes are injected individually with cRNA rather than transfected *en masse* with cDNA, one can be more assured that all the intended subunits are expressed in each, and in the intended quantities. However, no expression system is perfect, and *Xenopus* oocytes may lack some of the factors influencing *I*_to_ current characteristics in human heart, or endogenously express additional proteins not present in cardiac myocytes (although this criticism can also be leveled for immortal cell lines derived from mammals). Indeed, because of this, there are no claims herein that specific subunit combinations/splice variants better reflect native *I*_to_ than previous reports. Rather, a minimalist system has been utilized to uncover major, unanticipated Kv4.3 splice isoform-dependent functional differences at baseline. A comparison of different KChIP2 splice variants was not included here, and only the recently cloned long forms of KCNE3 and KCNE4 (Abbott, 2016c) were utilized, in favor of the short forms. In addition, other subunits that may also regulate Kv4.3 in human heart were not studied here. Thus, the experiments may fail to replicate other nuances that shape human cardiac *I*_to_. Neither were effects of Brugada syndrome-linked mutations in Kv4.3 (KCND3; Giudicessi et al., 2011), KCNE3 (Delpón et al., 2008) and KCNE5 (Ohno et al., 2011) quantified here in the context of isoform-specific effects, but it will be interesting to investigate potential differences in the future. Finally, effects of protein kinases were considered outside the scope of this study, but a subject for future work. An interesting contrast between the Kv4.3 isoform-dependent effects observed here compared to those previously reported for PKC-derived effects is that for the latter, Kv4.3L characteristics dominated when the two isoforms were co-expressed and PKC activated (Po et al., 2001); in the present study, Kv4.3S characteristics presided (and were apparently further augmented) when co-expressed with Kv4.3L, KChIP2b and KCNE4 (Figure 6).

## Author contributions

The author confirms being the sole contributor of this work and approved it for publication.

## Funding

This work was supported by University of California, Irvine School of Medicine setup funds.

### Conflict of interest statement

The author declares that the research was conducted in the absence of any commercial or financial relationships that could be construed as a potential conflict of interest.

## Abbreviations

HF: heart failure
KChIP: K^+^ channel interacting protein
Kv channel: voltage-gated potassium channel
PKC: protein kinase C.
